# Assessing descriptions of scalability for hypertension control interventions implemented in low-and middle-income countries: A systematic review

**DOI:** 10.1371/journal.pone.0272071

**Published:** 2022-07-28

**Authors:** Joyce Gyamfi, Dorice Vieira, Juliet Iwelunmor, Beverly Xaviera Watkins, Olajide Williams, Emmanuel Peprah, Gbenga Ogedegbe, John P. Allegrante

**Affiliations:** 1 Teachers College, Columbia University, New York, NY, United States of America; 2 NYU School of Global Public Health, New York, NY, United States of America; 3 NYU Health Sciences Library, New York, NY, United States of America; 4 NYU Grossman School of Medicine, NYU Langone Health, New York, NY, United States of America; 5 College for Public Health and Social Justice, Saint Louis University, Saint Louis, MO, United States of America; 6 Columbia University Medical Center, New York, NY, United States of America; 7 Mailman School of Public Health, Columbia University, New York, NY, United States of America; DM Wayanad Institute of Medical Sciences, INDIA

## Abstract

**Background:**

The prevalence of hypertension continues to rise in low- and middle-income- countries (LMICs) where scalable, evidence-based interventions (EBIs) that are designed to reduce morbidity and mortality attributed to hypertension have yet to be fully adopted or disseminated. We sought to evaluate evidence from published randomized controlled trials using EBIs for hypertension control implemented in LMICs, and identify the WHO/ExpandNet scale-up components that are relevant for consideration during “scale-up” implementation planning.

**Methods:**

Systematic review of RCTs reporting EBIs for hypertension control implemented in LMICs that stated “scale-up” or a variation of scale-up; using the following data sources PubMed/Medline, Web of Science Biosis Citation Index (BCI), CINAHL, EMBASE, Global Health, Google Scholar, PsycINFO; the grey literature and clinicaltrials.gov from inception through June 2021 without any restrictions on publication date. Two reviewers independently assessed studies for inclusion, conducted data extraction using the WHO/ExpandNet Scale-up components as a guide and assessed the risk of bias using the Cochrane risk-of-bias tool. We provide intervention characteristics for each EBI, BP results, and other relevant scale-up descriptions.

**Main results:**

Thirty-one RCTs were identified and reviewed. Studies reported clinically significant differences in BP, with 23 studies reporting statistically significant mean differences in BP (*p* < .05) following implementation. Only six studies provided descriptions that captured all of the nine WHO/ExpandNet components. Multi-component interventions, including drug therapy and health education, provided the most benefit to participants. The studies were yet to be scaled and we observed limited reporting on translation of the interventions into existing institutional policy (n = 11), cost-effectiveness analyses (n = 2), and sustainability measurements (n = 3).

**Conclusion:**

This study highlights the limited data on intervention scalability for hypertension control in LMICs and demonstrates the need for better scale-up metrics and processes for this setting.

**Trial registration:**

Registration PROSPERO (CRD42019117750).

## Introduction

Cardiovascular disease-related deaths in low- and middle-income countries (LMICs) is increasing rapidly [1] with hypertension expected to cause 7.5 million deaths per year [2]. Globally, the prevalence of hypertension is estimated at 40% [3], and continues to rise in LMICs where scalable, evidence-based interventions (EBIs) that are designed to reduce morbidity and mortality attributed to hypertension have yet to be fully adopted or disseminated. Multiple barriers at the systems, provider, and patient levels impede hypertension control [4, 5], which is complicated by low disease-related knowledge and literacy [5–8] and fragile healthcare systems. Failing to implement effective, evidence-based strategies will result in a projected 68% (125.5 million) increase in hypertension by 2025 [2] and unacceptably low rates of hypertension control in LMICs. Therefore, innovative approaches to curtail hypertension in LMICs are warranted and should be scaled both vertical and horizontal [9].

Scaling-up EBIs at all levels is necessary for effective long-term control of hypertension in LMICs. The WHO/ExpandNet, a global health network of public health professionals and scientists whose focus is to advance the practice and science of successful health innovations, defines scaling-up as “deliberate efforts to increase the impact of successfully tested health innovations so as to benefit more people and to foster policy and programme development on a lasting basis” [9]. However, optimal implementation and program expansion has received minimal attention [10]. Moreover, there is no uniform definition with which to measure scalability across studies [11].

Using the WHO/ExpandNet scale-up components as a guide, the objectives of this study were to evaluate evidence from published randomized controlled trials using EBIs for hypertension control implemented in LMICs and identify the WHO/ExpandNet scale-up components that are relevant for consideration in “scale-up” implementation planning.

## Methods

This review is registered in PROSPERO (CRD42019117750) https://www.crd.york.ac.uk/prospero/display_record.php?RecordID=117750.

### Search strategy

We developed a comprehensive search strategy to identify published trials that met predefined inclusion criteria using the Standard Cochrane Collaboration systematic review technique [12] and the Preferred Reporting Items for Systematic reviews and Meta-Analysis (PRISMA) [13]; World Bank criteria [14] were used to define LMICs. The search terms used were similar to those used by Ogedegbe et al. 2014 [4]. The final strategy included MeSH, Emtree and PsycINFO subject headings, keywords, and author-generated keywords (S1 File). The following databases were searched: Web of Science Biosis Citation Index (BCI), CINAHL, EMBASE, Global Health, Google Scholar, PubMed/Medline, and PsycINFO; the grey literature and clinicaltrials.gov were also searched. The initial search was conducted on July 7, 2018, and updated June 3, 2021.

### Inclusion and exclusion criteria

Studies were included if they: 1) were published RCTs implemented in LMICs, 2) reported on hypertension control intervention(s), 3) stated a primary and/or secondary outcome of change in blood pressure, 4) included “scale-up” or a variation of the term “scale,” and 5) were published in English. No limitation was placed on publication year or age of participants included in the study, and non-randomized studies, protocols, and systematic reviews were excluded.

### Data extraction

All citations were downloaded to EndNote and then exported to Google Sheets. Titles and abstracts of all articles were independently screened and rated (by JG and DV) to determine if they met inclusion criteria. Discrepancies regarding eligibility of studies were resolved by discussion between the two raters (J.G. and D.V.), and, if necessary, a third party. We then conducted full-text article review and extracted relevant information from the articles that met all of the study’s inclusion criteria. Specifically, the following study characteristics were retrieved and coded: country, setting, design, sample size, intervention type, duration, professional implementing the intervention, primary and or secondary blood pressure outcome, blood pressure findings, including mean systolic blood pressure (SBP) and diastolic blood pressure (DBP), mean difference in SBP and DBP between the treatment and control groups, and WHO/ExpandNet components discussed, with extraction of specific descriptions addressing each component. Identification of WHO/ExpandNet components [15] were mostly implied rather than explicitly stated. We applied the definition of each component (Table 1) to identify relevant information from each eligible article. Data were stored in Excel and analyzed with SPSS statistical software.

**Table 1:** Conceptual and operational definitions for scalability components.

Scalability Component	Description of Component	Key Questions
Input	Resources: human, material (facilities), equipment	Given that the intervention is proven effective, what resources are required to carry out and sustain the intervention broadly?
	(i.e., No. of nurses or other healthcare professionals trained, facilities, space, blood pressure monitors, etc.)	Is there a standard intervention implementation protocol across sites?
		Was staff adequately trained and or re-trained on study protocol?
Output	Services: access to more services, improving quality, efficiency, feasibility, fidelity	Is it a single intervention or multiple complex interventions?
		Will the intervention cover health insurance for patients, provide medications, pay for fruits and vegetables, etc.?
		Does staff have proficiency in what they are expected to do as part of the intervention?
		Random structured observations to assess the extent to which the intervention is being implemented as planned?
Outcome	Reaching individual clients: coverage; reaching groups/clients (utilization)	Are participants recruited only in the clinic or is there an outreach component (i.e., use of churches, salons, etc.)?
Impact	Benefits / lack of benefit as a result of using the intervention (i.e., decrease blood pressure among HTN patients)	Did the study address a persistent problem for the population? What was achieved? Did the study meet the objective? Did the study improve/worsen the health condition?
Equity	Fair and equal distribution of healthcare to those populations most in need	Does the study include both gender and or oversampling of the population at risk?
		Is there a plan to reach or recruit at risk individuals from rural areas/difficult to reach places?
Sustainability Measures	Sustainability- the likelihood that a project will continue to function effectively for the foreseeable future post project completion; Must have a "maximum" reach and integrated into the already existing healthcare system; Strong community and government support and resources are essential	Are the intervention benefits provided to the participants
		consistent over time?
		Is the intervention a stand-alone intervention or was it designed to be embedded into the existing healthcare system?
		Are components of the intervention compatible with the existing system?
		Are any of these implementation outcomes considered (feasibility, fidelity, penetration, acceptability, sustainability, uptake, and costs?
		Is there buy-in from the stakeholders?
		Did the study conduct any assessment post initial intervention period to check for sustainability?
Embed Within Current Health Organization Policy	Assess current health policy or organizational policy for treating condition and strategic aims and culture of the organizations involved	Is intervention aligned with the organizational goals?
		Infrastructure for intervention?
		Does organization and or investigative team have the capacity to carry out intervention?
		Does planned scale-up strategy fits with strategic aims and culture of the organization?
Costs/Cost Effectiveness	Assess the costs associated with the intervention and its cost effectiveness compared to its expected benefits	Is the intervention affordable in the current context?
		Is there an understanding of the resources needed to bring the intervention to scale?
		Are local financial resources set aside to accommodate future scale-up process?
Monitoring/ Evaluation	Assess the monitoring and evaluation process.	Is there an assurance that the intervention was implemented as planned (monitoring) in order to achieve desired results (evaluation)?
	Any statements relating to improving output, outcomes, impact, and Fidelity	

Note: Adapted from WHO/ExpandNet recommendations [15]

### Quality assessment methods

Risk of bias and quality of studies were assessed by two reviewers using the Cochrane Handbook for Systematic Review of Interventions, Version 5.1.0 [12] and the Cochrane risk-of-bias tool. Biases assessed included random sequence generation (selection bias), allocation concealment (selection bias), blinding of participants and personnel (performance bias), blinding of outcome assessment (detection bias), incomplete outcome data (attrition bias), and selective reporting (reporting bias). Quality of all trials was categorized as low/high/unclear risk of bias for each item mentioned above individually. Low risk of bias indicated that the item was well described and accounted for in the study; high risk of bias indicated the item was not sufficiently described in the study; and unclear risk of bias indicated that there was no information provided in the article to enable determination of the specific item of bias. All data were analyzed in Review Manager (RevMan 5.3).

## Results

**Fig 1:**
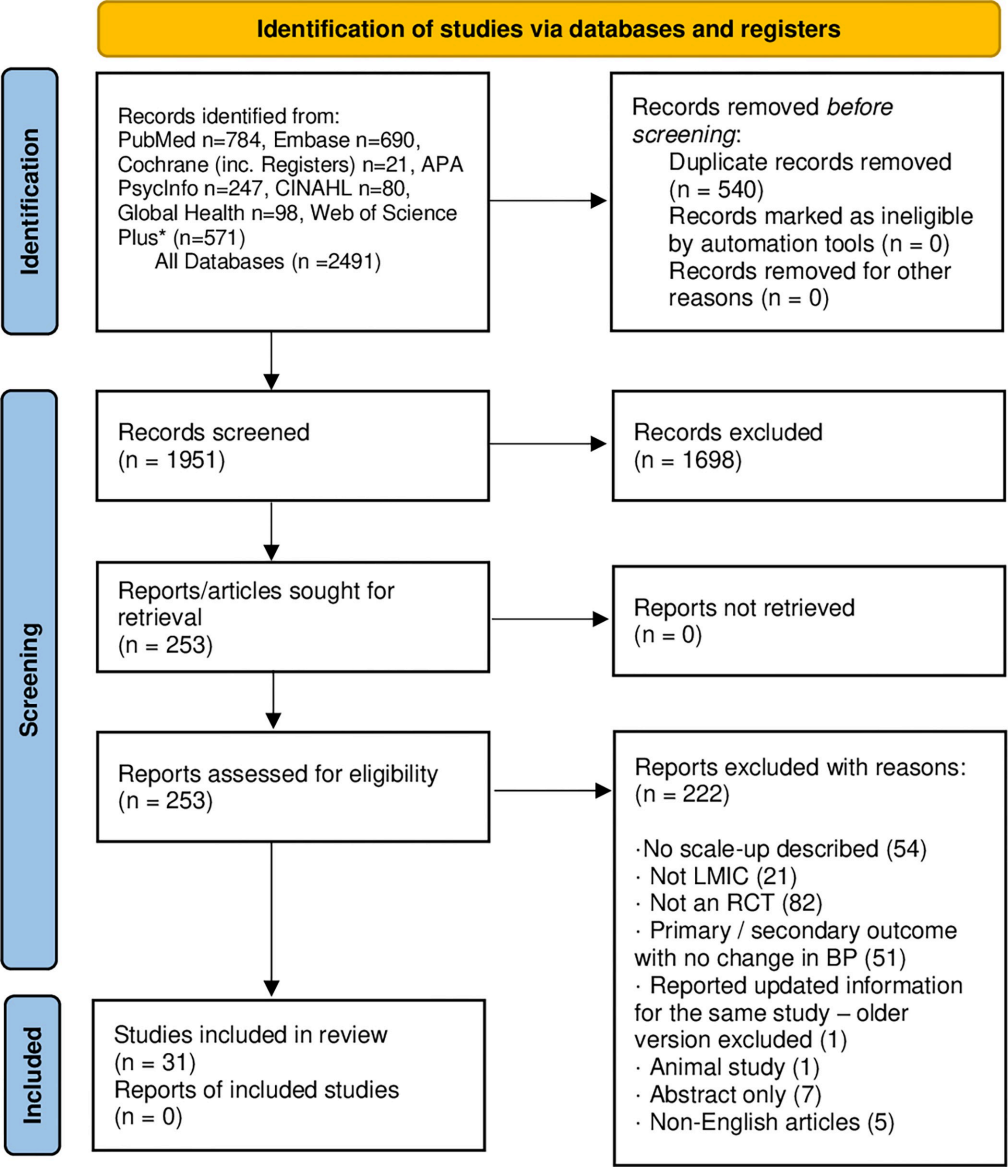
PRISMA flowchart of the selection of articles for the systematic review.

A total of 2,491 articles were identified (Fig 1). After removing duplicates, 1,951 titles and abstracts were independently screened. Of these, 1,698 articles were excluded, yielding 253 articles for which full texts were obtained and reviewed. During full-text review, 222 studies were excluded for the following reasons: No-scale-up plans described (n = 54), not conducted in LMICs (n = 21), not an RCT (n = 82), primary/secondary outcome was not change in blood pressure (n = 51), reported updated information for the same study–older version excluded (n = 1), animal study (n = 1), abstract only (n = 7), and non-English articles (n = 5). Thus, 31 papers met all study inclusion criteria and were eligible for the final review [3, 16–45]. All of the interventions reviewed were yet to be brought to full scale; however, the authors mentioned future plans for scale-up.

The majority of the studies (n = 19) were implemented mainly in South and East Asia, including Bangladesh (n = 1), Bangladesh, Pakistan, and Sri Lanka (n = 1), China (n = 9), India (n = 2), China and India (n = 1), Pakistan (n = 3),Vietnam (n = 1), and Mongolia (n = 1) (Table 2). The other studies were conducted in South America (Argentina n = 1; Brazil, n = 1; Chile, n = 1), sub-Saharan Africa (Ghana, n = 2), Nigeria and China (n = 1), Nigeria and Jamaica (n = 1), and the Middle East (Turkey, n = 5; Iran, n = 1)). The settings included: hospitals (n = 9), health centers/community setting (n = 16); urban settings (n = 18), rural settings (n = 5), both urban and rural settings (n = 2), and not reported (n = 6). Interventions were delivered by various health professionals, including physicians, nurse case managers, pharmacists, nutritionists, community health nurses, community health workers, and other primary healthcare workers.

**Table 2:** Systolic and diastolic blood pressure from RCT studies.

Authors (Year) and Country	Intervention	Duration of Intervention	Mean Difference in SBP (95% CI)	P-value	Mean Difference in DBP (95% CI)	P-value
Aira et al. (2013) Mongolia [16]	Health promotion [**Diet, Physical activity**] vs. financial literacy training	6 weeks	-0.04 (-3.24, 3.16)	0.389	0.84 (-1.98,3.66)	0.266
Bekiroğlu et al. (2013) Turkey [17]	Turkish Classical Music therapy vs. 25 minutes rest [**Music**]	28 days	-0.5	0.382	0	0.471
Chen et al. (2013) China [18]	Mindfulness Meditation vs. Pre-post measurements [**Meditation**]	7 days	-2.2	0.034*	-1	0.280
Danaoğlu et al. (2003) Turkey [19]	Statin + ACE inhibitor vs. ACE-inhibitor alone [**Medication**]	12 weeks	0	0.0001*	-0.1	0.0001*
Delavar et al. (2020) Iran [20]	HTN Self-Management [**Health** Literacy **Education** + **medication adherence]** vs. usual care	3 months		0.011*		0.029*
Forrester et al. (2005) Nigeria, Jamaica [21]	Low vs. High Salt intake	8 weeks	N (-4.5) (-1.6, -7.3); J (-5.5) (-8.0, -3.0)		N (-2.7) (-4.5, -0.9); J (-2.8) (-5.0, -0.5)	
Gamage et al. (2020) India [22]	CHW-led group-based **health education** and monitoring program [**medication adherence and lifestyle changes]** vs. usual care	3 months	−5.0 (−7.1, −3.0)	<0.001*	−2.1 (−3.6, −0.6)	<0.006 *
Gong et al. (2018) China [23]	Healthy Heart and Healthy Brain (KM2H2) Physical Activity plus standard care vs. standard care [**Health Education, Physical activity, Counseling**]	6 months	-3.02	0.21	-1.6	0.370
Hacihasanoğlu et al. (2011) Turkey [24]	Education medication adherence (Group A) vs. Education in medication compliance plus Healthy lifestyle behavior education [**Medication adherence, Physical activity, Diet, Weight loss, Stress management**] (Group B) vs. Pre-post measurements (Group C)	6 months	-22.5	< 0.001*	-2.25	< 0.001*
He et al. (2017) Argentina [25]	Health coaching, home BP monitoring and audit and feedback, text messaging [**Health Education**] vs. Usual care	18 months	-6.6 (-4.6, -8.6)	< 0.001*	-5.4 (-4.0,-6.8)	< 0.001*
Huang et al. (2018) China [26]	SMS (Self-management education and MI) vs. standard care and routine health education [**Health Education**]	5 weeks	-3.3 (-9.7,-3.0)	0.2901	-4.7 (-8.7,-1.1)	0.0133*
Huang et al. (2012) China [27]	Zolpidem vs. placebo [**Medication**]	30 days	-7.4	< 0.05*	-3.9	< 0.05*
Jafar et al. (2009) Pakistan [29]	Family-based HHE delivered by community health workers and GP education with a case-based curriculum for blood pressure management [**Health Education**] vs. No intervention	24 months	-10.8 (-8.9, -12.8)	< 0.001*	-5.8 (3.9 to 7.7)	< 0.001*
Jafar et al. (2010) Pakistan [30]	Community home health education on BP [**Diet, Physical Activity, Weight loss, Tobacco cessation**] vs. No home health education	3 months	1.4	0.02*	1.5	0.002*
Jafar et al. (2020) Bangladesh, Pakistan, and Sri Lanka [28]	Home visits by trained CHW for BP monitoring and counseling, training of physicians, and care coordination in the public sector [**Health Education, Training, Referral**] vs. Existing community services	24 months	-5.2 (-3.2, -7.1)	<0.001*	-2.8 (-1.7, -3.9)	
Khan et al. (2019) Pakistan [31]	Both arms had enhanced screening and diagnosis of hypertension and related conditions, and patient recording processes. Intervention facilities also had a clinical care guide, additional drugs for hypertension, a patient lifestyle education flipchart, associated training, and mobile phone follow-up. [**Health Education, Medication Adherence**]	9 months.	-12.63 (- 0.68, -24.57)	0.04*	-7.58 (-0.61, -14.55);	0.04*
Kolcu et al. (2020) Turkey [32]	Nurse-led hypertension management program [**Health Education, Motivational meetings, Health Behavioral counselling**] vs. Routine HTN care in nursing home	5 months	-7.84	0.000*	-11.62	0.000*
Li et al. (2009) China [34]	Reduced sodium, high-potassium salt substitute vs. Normal salt	12 months	-	-	-	-
Li et al. (2010) China [33]	Chinese Medicine (CM) vs. Western Medicine vs. Combination [**Medication**]	4 weeks	-3.33	< 0.05*	-0.36	> 0.05*
Li, X et al. (2019) China [44]	Health education, health promotion, group chat, and blood pressure (BP) monitoring [**Health Education and Health promotion**] vs. usual community health services	6 months	−6.9 (−11.2, −2.6)	0.002*	-3.1 (−5.7, −0.6	0.016*
Lin et al. (2014) China [35]	Text messaging-assisted lifestyle weight loss intervention vs. Brief information session [**Health education, Diet, Physical Activity, Weight loss]**	6 months	-4.14 (-0.98,-7.29)	0.01*	-4.43 (-2.00,-6.87)	0.0004*
Ma et al. (2014) China [45]	Motivational interviewing [**Diet, Physical activity, Medication adherence, Alcohol intake reduction, Smoking cessation, Stress management**] vs. Usual care	6 months	-4.92	0.011*	-2.58	0.027*
Mendis et al. (2010) China, Nigeria [3]	WHO CVD package, Hydrochlorothiazide, lifestyle counseling [**Medication plus health education**] vs. Usual care	12 months	Site A (-3.86); Site B (-4.4)	< 0.05*	Site A (-1.53); Site B (-3.33)	-
Naser et al. (2020) Bangladesh [36]	Drinking managed aquifer recharge water on blood pressure and urine protein among study participants vs. brackish groundwater-drinkers	5 months	-1.33 (-0.32, -2.34)	0.010*	-0.64 (-0.20, -1.48	0.136
Nguyen et al. (2018) Vietnam [37]	Stories in the patients’ own words about coping with hypertension and didactic content about the importance of healthy lifestyle behaviors in con- trolling elevated blood pressure levels [**Health Education**] vs. Didactic content about HTN & other NCD management	12 months	-2.7 (-3.0,-8.4)	-	-0.9	-
Ogedegbe et al. (2018) Ghana [38]	Task-shifting strategy + Health Insurance vs. Health insurance only [**Health Insurance, Health Education, Behavioral Counseling, Medication]**	12 months	-3.6 (-6.1, -0.5)	0.021*	-1.2 (-2.4, 1.2)	-
Pedrosa et al. (2013) Brazil [39]	Standard HTN treatment plus CPAP vs. Standard HTN treatment [**Medication**]	6 months	-9.6	< 0.05*	-6.6	< 0.05*
Sarfo et al. (2019) Ghana [40]	Blue-toothed BP device and smartphone with an App for monitoring BP measurements and medication intake under nurse guidance [**Health Education plus medication**] vs. usual care	9 months	-	0.035*	-	0.03*
Tian (2015) China, India [41]	Simplified Cardiovascular management program delivered by community health workers with the aid of a smartphone electronic decision support system [**Health Education plus medication**]vs. Usual care	24 months	-2.7	0.04*	-	-
Varleta et al. (2017) Chile [42]	Text messaging [**Diet, Salt intake, Medication intake, and adherence**] vs. No text messaging	6 months	-	-	-	-
Yilmaz et al. (2011) Turkey [43]	Alprazolam vs. Captopril[**Medication]**	2 hours	0.36	0.626	-	-

Note: (-) before mean difference denotes reduction

* = *p* < 0.05; information not reported (-)

### Interventions

Of the RCTs reviewed, the interventions reported included diet, physical activity, medication, behavioral lifestyle counselling, and/or a combination of health education and health promotion strategies (Table 2). Duration of intervention lasted from less than one month to 36 months, with the exception of the Turkish medication study, which lasted two hours [43]. The interventions provided clinically significant reduction in blood pressure for study participants. The studies conducted in Pakistan and Chile, used salt reduction strategy, lifestyle counselling, and health education through text messaging, respectively; however, the mean difference in SBP and/or DBP or the significance was not reported [34, 42]. The primary or secondary outcomes of all studies included change in SBP and/or DBP. Sample size ranged from 39 to 4,023 participants.

The trial participants were adults over 18 years of age, with equal distribution of males and females. Eleven studies reported mean participant income: six studies, low income ($1,025 or less/month) [16, 21, 29, 30, 38, 40]; four studies, low-middle income ($1,026-$4,035/month) [18, 20, 22, 35]; and one upper-middle income ($4,036 - $12,475) [32]. The dominant study design employed was parallel design (n = 16), with the remaining studies using a cluster design (n = 13), cross-over design (n = 1) and step-wedge (n = 1). All of the studies used non-probability sampling, with the exception of five studies conducted in Brazil, China, China/India, Vietnam, and Turkey [18, 24,37, 39, 41].

Only four studies [3, 17, 25, 38] involved local or national stakeholders. Two of these consulted local health officials to guide the study site selection in Nigeria and China, and Ghana [3, 38]; one consulted traditional music specialists in the community prior to the music selection for the intervention [17]; and one study selected health centers in Argentina based on expert recommendations from the country’s national public health system [25].

### Interventions and blood pressure control

The majority of the 31 studies reported clinically significant decreases in SBP and DBP among participants exposed to the intervention (Table 2). Multicomponent interventions comprising education about healthy lifestyle (i.e., diet, physical activity, etc.), providing antihypertensive medications or education about medication adherence, and implemented over a longer duration (6–12 months) were more effective in reducing blood pressure. Moreover, 23 (74%) of the studies found a statistically significant mean difference in SBP and/or DBP (*p* < 0.05) with the greatest mean SBP and DBP difference of -22.5 mmHg (*p* < 0.001) and 11.62 mmHg (*p* < 0.000), respectively [24, 31]. The studies that did not reach statistical significance were single-focused interventions (diet, physical activity, or medication solely), and/or were of shorter duration (2 hours to 6 months) [17, 23, 43]. The majority of the studies reported using automated machines to measure blood pressure, while others utilized a manual mercury sphygmomanometer.

### Descriptions of evidence of scalability components

Of the 31 articles that mentioned a plan for scale-up, the majority briefly described the need for expansion or replication (horizontal scaling-up). As displayed in Table 3, only two studies provided descriptions suggestive of policy, political, legal, regulatory, budgetary, or other health systems changes necessary to institutionalize the interventions at a national level (vertical scale-up) [16, 38]. One study [38] reported future plans for horizontal and vertical scale-up, which included a cost-effectiveness evaluation of the task-shifting strategy for hypertension control in various health centers and community-based health planning and service (CHPS) compounds in Ghana, and its potential scale-up across Ghana and other countries in Sub-Saharan Africa, while engaging policymakers. Another study [16] described scale-up as the feasibility of implementation; large-scale disease prevention; and engagement of private and public interest, political support, and policy and administrative institutions that can initiate, implement, sustain, and evaluate such programs for improving the long-term health of the population.

**Table 3:** Summary of RCTs and scalability of hypertension interventions implemented in low- and middle-income countries.

Authors and Country	RCTDesign/Type	Sample Size	Intervention	Duration of Intervention	Description of Scalability	No. of WHO/ ExpandNet Components Addressed
Aira et al. (2013) Mongolia [16]	Parallel	200	Health promotion [**Diet, Physical activity**] vs. financial literacy training	6 weeks	Feasibility of implementation, large-scale disease prevention, engagement of private and public interest	9
Bekiroğlu et al. (2013) Turkey [17]	Parallel	60	Turkish Classical **Music therapy** vs. 25 mins. rest	28 days	Improve clinical practice	7
Chen et al. (2013) China [18]	Parallel	60	Mindfulness **Meditation** vs. Pre-post measurements	7 days	Reduce illness and disease prevention (impact)	6
Danaoğlu et al. (2003) Turkey [19]	Parallel	39	Statin + ACE inhibitor vs. ACE-inhibitor alone [**Medication**]	12 weeks	Long-term large-scale studies for effectiveness clarification	6
Delavar et al. (2020) Iran [20]	Parallel	118	HTN Self-Management **Health Literacy Education** + **medication adherence** vs. usual care	3 months	Future studies could be conducted to assess the effectiveness of SME based on HLI on other chronic conditions	6
Forrester et al. (2005) Nigeria, Jamaica [21]	Cross-over	114	Low salt vs. High salt intake [53]	8 weeks	Sustainment of intervention overtime to decrease burden of disease	8
Gamage et al. (2020) India [22]	Cluster	1,734	CHW-led group-based **health education** and monitoring program [**medication adherence and lifestyle changes]** vs. usual care	3 months	Country-wide scale-up to diverse rural settings and to other resource-poor regions in other countries	7
Gong et al. (2018) China [23]	Cluster	450	Healthy Heart and Healthy Brain (KM2H2) Physical Activity plus standard care vs. standard care [**HTN education, Physical activity, Counseling**]	6 months	Larger samples to assess effectiveness while emphasizing objective measures, rigorous protocol, and training of health professionals (input)	7
Hacihasanoğlu et al. (2011) Turkey [24]	Parallel	120	Education in medication adherence (Group A) vs. Education in medication compliance plus Healthy lifestyle behavior education [**Medication adherence, Physical activity, Diet, Weight loss, Stress management**] (Group B) vs. Pre-post measurements (Group C)	6 months	Global application/ expansion to other primary care facilities	8
He et al. (2017) Argentina [25]	Cluster	1,432	Health coaching, home BP monitoring and audit and feedback, text messaging [**Health Education**] vs. Usual care	18 months	Widespread scaling-up of this proven effective intervention in LMICs should result in controlled hypertension and reduce related cardiovascular disease	9
Huang et al. (2018) China [26]	Parallel	83	CPAP vs. No therapy [**Medication**]	36 months	Larger samples to clarify impact	6
Huang et al. (2012) China [27]	Parallel	90	SMS (Self-management education and MI) vs. standard care and routine health education [**Health Education**]	5 weeks	Sustain healthy behaviors and improve BP control	6
Jafar et al. (2009) Pakistan [29]	Cluster	1,341	Family-based HHE delivered by community health workers and GP education with a case-based curriculum for blood pressure management [**Health Education**] vs. No intervention	24 months	Adaptation of intervention to other resource-poor settings while monitoring context effectiveness to ensure full integration into existing health care systems of developing countries	8
Jafar et al. (2010) Pakistan [30]	Cluster	4,023	Community home health education on BP [**Diet, Physical activity, Weight loss, Tobacco cessation**] vs. No home health education	3 months	Evaluation of cost effectiveness, human resources, and training	7
Jafar et al. (2020) Bangladesh, Pakistan, and Sri Lanka [28]	Cluster	2,645	Home visits by trained CHW for BP monitoring and counseling, training of physicians, and care coordination in the public sector [**Health Education, Training, Referral**] vs. Existing community services	24 months	Scale up might translate into substantial reductions in premature deaths and disability, as well as social and economic returns	9
Khan et al. (2019) Pakistan [31]	Cluster	1,138	Both arms had enhanced screening and diagnosis of hypertension and related conditions, and patient recording processes. Intervention facilities also had a clinical care guide, additional drugs for hypertension, a patient lifestyle education flipchart, associated training, and mobile phone follow-up. [**Health Education, Medication Adherence**]	9 months	Scaling of an integrated CVD–hypertension care intervention in urban private clinics in areas lacking public primary care in Pakistan	8
Kolcu et al. (2020) Turkey [32]	Parallel	74	Nurse-led hypertension management program [**Health Education, Motivational meetings, Health Behavioral counselling**] vs. Routine HTN care in nursing home	5 months	Participants maintaining behavioral modifications after program	6
Li et al. (2009) China [34]	Parallel	608	Reduced sodium, high-potassium salt substitute vs. Normal salt [53]	12 months	Widespread acceptability of intervention	7
Li et al. (2010) China [33]	Parallel	241	Chinese Medicine (CM) vs. Western Medicine vs. Combination [**Medication**]	4 weeks	Large-scale RCTs to reduce cardiovascular or all-cause mortality	5
Li, X et al. (2019) China [44]	Cluster	464	Health education, health promotion, group chat, and blood pressure (BP) monitoring[**Health Education and Health promotion**] vs. usual community health services	6 months	Expansion of the intervention to the whole country with considerations of economic, technological, and medical developments	7
Lin et al. (2014) China [35]	Parallel	123	Text messaging-assisted lifestyle weight loss intervention trial vs. brief information session [**Health education, Diet, Physical Activity, Weight loss]**	6 months	Feasibility of implementation, reaching additional people	7
Ma et al. (2014) China [45]	Parallel	120	Motivational interviewing [**Diet, Physical activity, Medication adherence, Alcohol intake reduction, Smoking cessation, Stress management**] vs. Usual care	6 months	Placing intervention in context with training strategy, characteristics of the patients, and healthcare professionals	8
Mendis et al. (2010) China, Nigeria [3]	Cluster	2,397	WHO CVD package, Hydrochlorothiazide, lifestyle counseling [**Medication plus Health Education**] vs. Usual care	12 months	Standardize protocol implementation in large proportions of participants	9
Naser et al. (2020) Bangladesh [36]	Step-wedge	1,191	Drinking managed aquifer recharge water on blood pressure and urine protein among study participants vs. brackish groundwater-drinkers [53]	5 months	Scale up of new MAR system to reach additional populations	8
Nguyen et al. (2018) Vietnam [37]	Cluster	160	Stories in the patients’ own words about coping with hypertension and didactic content about the importance of healthy lifestyle behaviors in con- trolling elevated blood pressure levels [**Health Education**] vs. Didactic content about HTN & other NCD management	12 months	A large-scale randomized trial to systematically compare the short and long-term effectiveness of the two interventions	8
Ogedegbe et al. (2018) Ghana [38]	Cluster	757	Task-shifting strategy + Health Insurance vs. Health insurance only [**Health Insurance, Health Education, Behavioral Counseling, Medication**]	12 months	Application of a reliable strategy to other regions in Ghana and other SSA countries; incorporating a delivery of the intervention as part of the duties of nurses within existing healthcare system	9
Pedrosa et al. (2013) Brazil [39]	Parallel	35	Standard HTN treatment plus CPAP vs. Standard HTN treatment [Medication]	6 months	Impact of intervention on other people with other CVD outcomes	6
Sarfo et al. (2019) Ghana [40]	Cluster	60	Blue-toothed BP device and smartphone with an App for monitoring BP measurements and medication intake under nurse guidance [**Health Education plus medication**] vs. usual care	9 months	Larger scale studies to measure clinical outcomes related to hypertension control, whilst adapting the intervention to the local context	8
Tian (2015) China, India [41]	Cluster	2086	Simplified Cardiovascular management program delivered by community health workers with the aid of a smartphone electronic decision support system [**Health Education plus medication**]vs. Usual care	24 months	Scale up in more regions and other countries to benefit a large number of disadvantaged populations; and larger context specific trials to refine program and assess cost-effectiveness	9
Varleta et al. (2017) Chile [42]	Parallel	314	Text messaging [**Diet, Salt reduction, Medication intake and adherenc**e] vs. No text messaging	6 months	Maintenance of long-term effects in other patients	8
Yilmaz et al. (2011) Turkey [43]	Parallel	53	Alprazolam vs. Captopril [**Medication**]	2 hours	Repetition of intervention in other healthcare settings	5

**Note:** The papers were published between 2003 and 2021, with most studies published within the past 10 years.

The remaining studies described scalability using the following phrases: large scale improvement of clinical practice; reduce illness and disease prevention (impact); sustainment of intervention over time to decrease burden of disease; larger samples to assess effectiveness; emphasizing objective measures, rigorous protocol, and training of health professionals; adaptation of the intervention and integration into the existing health care system, widespread acceptability of intervention; maintenance of long-term effects in other patients; repetition of intervention in other healthcare settings; global application/expansion to other primary care facilities; human resources, training, and consideration of other operative expenses that can potentially impact spread. Among the studies with clear descriptions of scalability, the descriptions were based on WHO/ExpandNet components of scalability. On average, studies described a range of five to nine scalability components, with the majority focusing more on patient outcomes and horizontal scale-up than on the vertical scale-up.

### WHO/ExpandNet scale-up components

Table 4 summarizes the WHO/ExpandNet scalability components reported by the studies.

**Table 4:** WHO/ExpandNet components included in studies.

STUDIES	WHO/EXPANDNET SCALE-UP COMPONENTS								
AUTHORS (YEAR) / COUNTRY	INPUT	OUTPUT	OUTCOME	IMPACT	EQUITY	EMBEDDED WITHIN CURRENT HEALTH ORGANI-ZATION POLICY	COST/COST-EFFECTIVE-NESS	MONITORING/ EVALUATION	SUSTAIN-ABILITY
Aira et al. (2013)/ Mongolia [16]	X	X	X	X	X	X	X	X	XX
Bekiroğlu et al. (2013)/Turkey [17]	X	X	X	X	X	X		X	
Chen et al. (2013)/ China [18]	X	X	X	X	X			X	
Danaoğlu et al. (2003)/Turkey [19]	X	X	X	X	X			X	
Delavar et al. (2020)/Iran [20]	X	X	X	X	X			X	
Forrester et al. (2005)/Nigeria, Jamaica [21]	X	X	X	X	X	X		X	X
Gamage et al. (2020)/ India [22]	X	X	X	X	X		X	X	
Gong et al. (2018)/ China [23]	X	X	X	X	X			X	XX
Hacihasanoğlu et al. (2011)/Turkey [24]	X	X	X	X	X		X	X	X
He et al. (2017)/ Argentina [25]	X	X	X	X	X	X	XX	X	X
Huang et al. (2018)/ China [26]	X	X	X	X	X				
Huang et al. (2012)/ China [27]	X	X	X	X	X			X	
Jafar et al. (2009)/Pakistan [29]	X	X	X	X	X		X	X	X
Jafar et al. (2010)/ Pakistan [30]	X	X	X	X	X	X			X
Jafar et al. (2020)/Bangladesh, Pakistan, and Sri Lanka [28]	X	X	X	X	X	X	XX	X	XX
Khan et al. (2019)/ Pakistan [31]	X	X	X	X	X	X		X	X
Kolcu et al. (2020)/Turkey [32]	X	X	X	X	X			X	
Li et al. (2009)/ China [34]	X	X	X	X	X		X		X
Li et al. (2010)/ China [33]	X	X	X	X	X				
Li, X et al. (2019) China [44]	X	X	X	X	X			X	X
Lin et al. (2014)/ China [35]	X	X	X	X	X			X	X
Ma et al. (2014)/ China [45]	X	X	X	X	X	X		X	X
Mendis et al. (2010)/China, Nigeria [3]	X	X	X	X	X	X	X	X	X
Naser et al. (2020)/ Bangladesh [36]	X	X	X	X	X		X	X	X
Nguyen et al. (2018)/Vietnam [37]	X	X	X	X	X		X	X	X
Ogedegbe et al. (2018)/Ghana [38]	X	X	X	X	X	X	X	X	XX
Pedrosa et al. (2013)/Brazil [39]	X	X	X	X	X			X	
Sarfo et al. (2019)/Ghana [40]	X	X	X	X	X		X	X	X
Tian (2015)/China, India [41]	X	X	X	X	X	X	X	X	X
Varleta et al. (2017)/Chile [42]	X	X	X	X	X		X	X	X
Yilmaz et al. (2011)/Turkey [43]	X	X	X	X	X				

Note: XX = studies that reported findings from their cost-effectiveness analysis or sustainability assessment. The authors of the studies reviewed did not explicitly state the components in the articles. Therefore, we relied on statements provided in the paper that fit the description of each component.

#### Input

All of the articles reported on material and equipment inputs. Moreover, some studies described in detail the training provided to health professionals implementing the intervention. Twenty-one articles provided information about health professional training, including training in the study protocol, standardization of blood pressure measurements, motivational interviewing, lifestyle behavioral counseling, interview procedures, and data collection forms.

#### Output

The majority of the services offered included health promotion and health education (n = 19), medication therapy (n = 12), physical activity (n = 6), music therapy (n = 1), and meditation (n = 1). Eighteen studies used multicomponent interventions provided to participants over a duration ranging from <1 day to 36 months. Baseline and follow-up visits occurred every three months in most cases over the course of the treatment to check for intervention effects.

#### Outcome

All studies described outcomes. In ten studies, multisite (hospitals, health centers, and community) recruitment strategies were used to ensure that all patients who were eligible were screened. Studies focusing on a specific population were limited to a certain recruitment site (e.g., hospital or nursing home). Across the studies, sample size ranged from 17 to 4,023 participants, aged 18 years and over.

#### Impact

Twenty-three studies (74%) reported statistically significant mean differences in SBP and/or DBP between the intervention and the control participants.

#### Equity

Most studies were implemented in urban settings (n = 18), rural settings (n = 5), both urban and rural settings (n = 2), six studies did not provide sufficient information to determine the setting.

#### Embedded within current health organization policy

Only 11 out of 31 articles (35%) provided this information. One study [3] stated consulting the local health officials for the selection of health facilities, and included sites that had the infrastructure, resources, and healthcare staff to implement the protocol effectively. Similarly, in another study [38], Ghana’s CHPS program was used as a platform to engage community health nurses already employed at the health facilities to participate in the intervention implementation process. The other articles discussed matching components of their program with existing flow of care, integrating the new intervention into the facilities’ disease management system, and amending existing policy to accommodate new interventions.

#### Costs/Cost effectiveness

Only 14 (45%) studies mentioned cost or cost effectiveness of the intervention and/or future plans to assess the interventions’ cost effectiveness. Authors discussed assessing the cost of intervention(s) (i.e., affordability of fruits and vegetables and/or medications for the population), cost-effectiveness compared to other interventions, cost of medication, and suggested future studies to assess cost-effectiveness of the intervention. However, only two studies conducted a cost-effectiveness analysis [25, 28].

#### Monitoring/Evaluation

Twenty-six studies included monitoring of medication adherence through pill counts, frequent documentation of adverse events, use of patient diaries, patient self-reported intervention engagement, and case management.

#### Sustainability

Twenty-two (71%) of the articles referenced sustainability (e.g., uptake and maintenance of intervention, sustained decrease in post-trial blood pressure). However, only three studies assessed sustainability post-intervention delivery: the TASSH study in Ghana demonstrated sustainability of intervention effects 12 months post-trial implementation [38]; a study in Mongolia assessed continuation of the intervention three months post-intervention conclusion [16]; and a study in China found that participants continued and sustained their physical activity regimen 30 months post-intervention [23].

Overall, six studies provided descriptions that documented all nine WHO/ExpandNet components [3, 16, 25, 28, 38, 41]. Few studies focused on the importance of embedding the intervention into an already established healthcare system or a consideration of costs and cost-effectiveness; only 13% of the studies assessed post-implementation sustainability.

#### Risk of bias assessment

**Fig 2:**
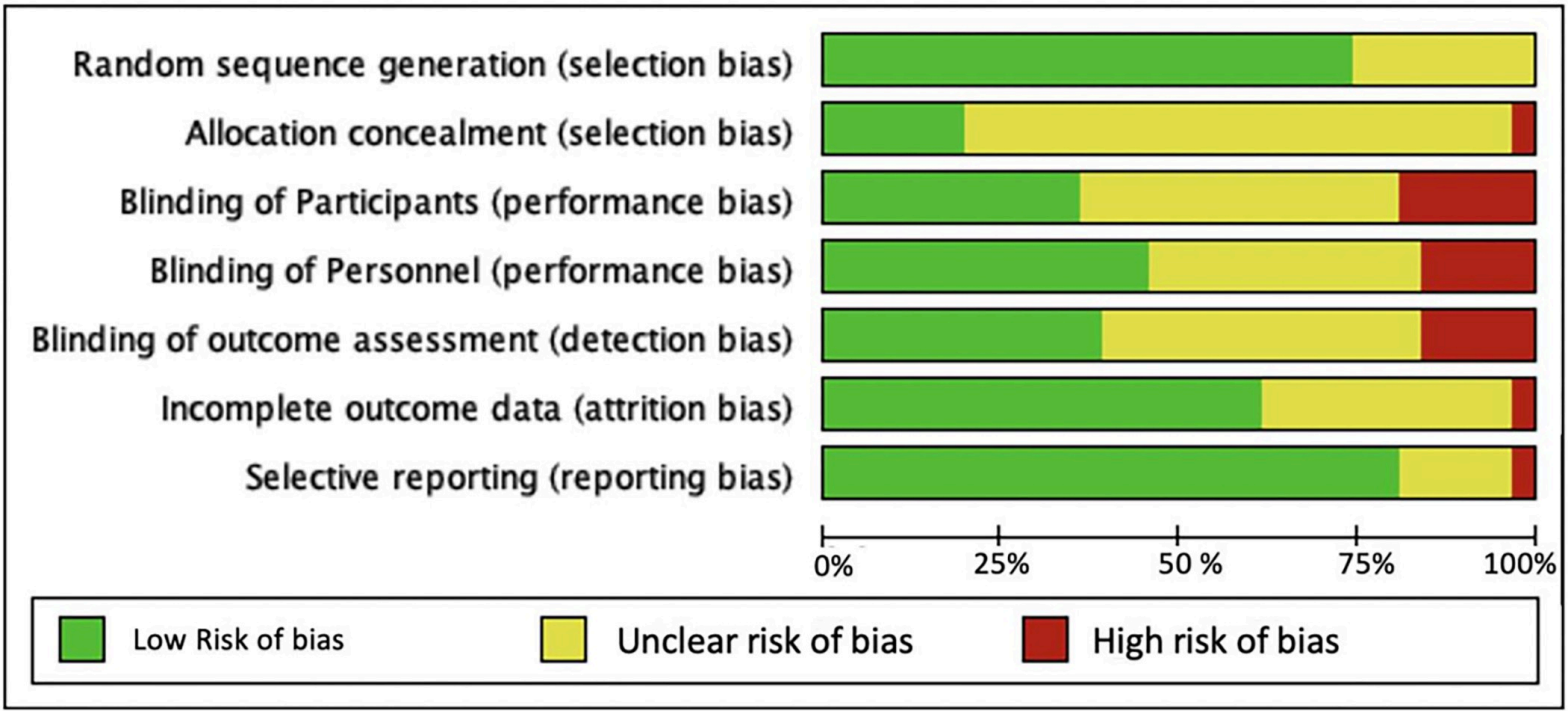
Risk of bias graph. Review authors’ judgments about each risk of bias item presented as percentages across all included studies (n = 31 studies).

**Fig 3:**
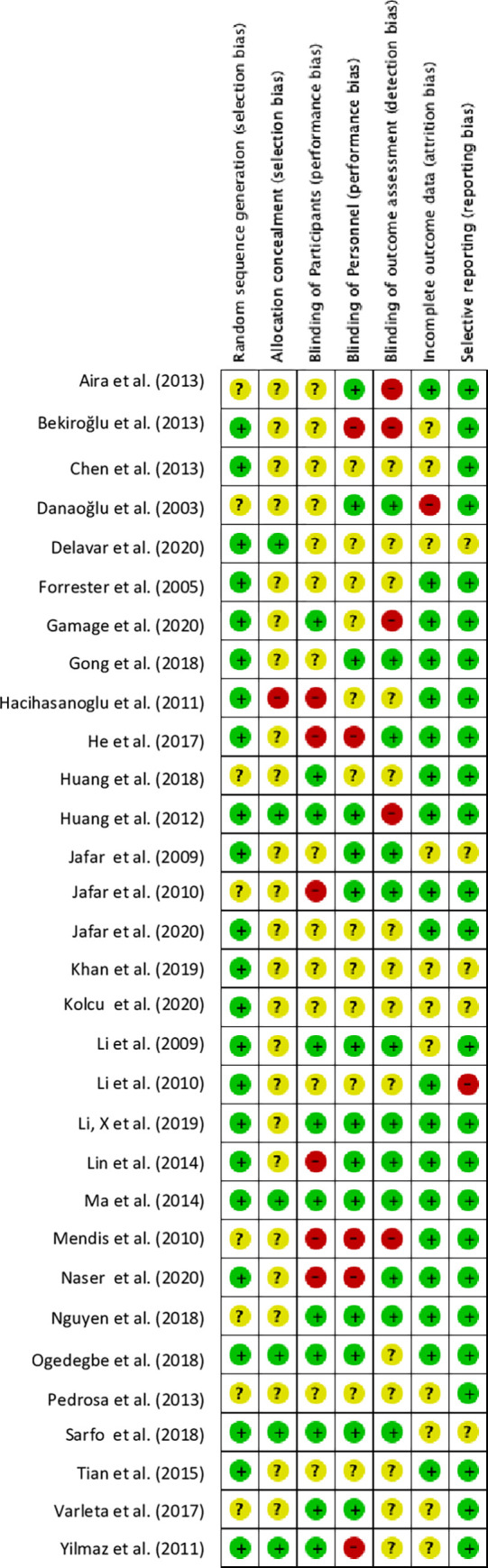
Risk of bias table. Review authors’ judgments about each risk of bias item for each article (n = 31 studies).

Based on the Cochrane Risk of Bias criteria [12] most studies (~75%) were classified as low risk of bias for random sequence generation (selection bias), and Selective reporting (reporting bias); however, over 70% of the studies did not provide information on allocation concealment (Figs 2 and 3). Approximately, 25 percent of the studies were rated high risk of bias for blinding of participant, personnel, and outcome assessment, mainly due to the nature of the study interventions not allowing for blinding to be possible. Participant withdrawal or dropout was reported by 19 studies (61%), and all of the trials reported following up the participants for at least two time points throughout the intervention.

## Discussion

We assessed the descriptions of scalability from published RCTs of EBIs for hypertension control in LMICs. Although most of the studies highlighted the need for horizontal scale-up, only two studies [16, 38] clearly described plans for scale-up using the WHO/ExpandNet recommended vertical and horizontal scale-up strategy. The WHO/ExpandNet scale-up recommendations were not available until 2010, therefore older studies could not reference the recommendations because they did not exist at the time of this review.

Overall, most of the studies focused on reporting patient outcomes rather than delineating plans for the scale-up process. This is consistent with one systematic review of 14 studies conducted in primary care settings in 11 LMICs and three HICs for the prevention of mostly infectious diseases [46]. The WHO/ExpandNet components that were frequently described included Inputs, Outputs, Outcomes, Impacts, Equity, Monitoring, and Evaluation; however, few studies focused on the importance of embedding the study into an already established healthcare system or on cost effectiveness and sustainability, with only two studies [25, 28] providing cost-effectiveness data. Furthermore, despite the need for EBI scale-up in LMICs, lack of capacity due to fragile health care systems as a result of limited human and material resources, and inadequately trained health professionals in implementation science may hinder the scale-up process.

Several observations emerge from this review. First, multicomponent interventions, including those comprising medications and health education for lifestyle behavioral changes (i.e., physical activity, diet, weight loss, smoking cessation), proved effective in decreasing blood pressure. The decrease in SBP ranged from -0.04 to -22.5 mmHg, and the mean difference for DBP ranged from -0.1 to 11.62 mmHg across all studies. Our findings are consistent with previous reviews showing that multicomponent intervention integrated into an already existing system, and that is tailored to the practice context [47], can be an effective means to improve hypertension control [48].

Second, incorporating EBIs into the regular operations of the healthcare system is key to scaling up. This eliminates the prospect of segmented care and increases the possibility of getting greater buy-in from stakeholders and the professionals implementing the intervention [49]. Incorporating health education as part of the intervention into an established system is effective for lowering blood pressure and long-term disease prevention [24, 30]. Furthermore, adapting the intervention to the changing context and in response to input from the leadership within an organization, throughout the process of development to implementation, is likely to foster greater support and ensure program adoption and sustainability. Individual, institutional, and systemic capacity building [50] including training of medical/public health researchers on scale-up approaches in resource-constrained settings is paramount.

Third, an understanding of the challenges LMICs face in scaling up effective interventions for the [51, 52] management of hypertension is critical to improving physician and healthcare worker practice; and to improving both patient satisfaction and health outcomes. Human resource, health system capacity, and stakeholder acceptance of intervention is key to successful scale-up. Further, building capacity for health professionals to be trained in scale-up of EBIs is a desired outcome in implementation science to advance intervention reach and adoption.

Fourth, considering the lack of a clear definition of scale-up and inconsistency in the descriptions of scale-up components provided in the articles reviewed, we propose the following definition of scale-up: “Widespread expansion and or replication of an evidence-based, context-tailored health interventions for various population groups with guaranteed human and material resources, while engaging all key stakeholders (i.e., patients, community members, providers, health care policy makers), throughout the implementation process to ensure sustainability.” This definition draws on existing definitions while incorporating elements that include context-specific interventions, availability of resources, continuous stakeholder involvement at all levels, and sustainability considerations. Moreover, interventions being scaled in the context of low resource settings should be cost-effective; and implementation should be sensitive of the cost burden to the setting in relation to health outcomes. Finally, we recognize that the WHO/ExpandNet guides are designed for actual scale-up studies, however, using it as a guide to identify scale-up components of studies yet to be scaled will provide guidance to implementers for successful scale-up efforts. Scale-up should be considered early, preferably during intervention planning and protocol design; and descriptions of the WHO/ExpandNet components should include sufficient details to enable replication. Concerted efforts should be made to improve the reporting of conceptualization, operationalization, and measurement of scalability in published literature by drawing on the terminologies used in the implementation science literature.

This review has some limitations. First, we assessed only RCTs that were published in English. Second, studies using designs other than RCT were not included, which may also have scalability descriptions, and therefore for future research, we recommend a scoping review of non-RCTs to capture other scalable interventions for hypertension control reported elsewhere. Finally, because of heterogeneity in terms of treatment and comparators across studies, we were unable to obtain the pooled means for SBP and DBP, and a meta-analysis of the effect sizes was not performed. Finally, the conclusions should be interpreted with some caution due to the lack of a clear definition of scale-up across studies.

Despite these limitations, we believe the review has several important strengths. We conducted a rigorous systematic search, using *a priori* inclusion and exclusion criteria to retrieve articles that reported studies using EBIs for hypertension control in LMICs across multiple databases and assessed the WHO/ExpandNet scale-up components reported within the studies that met the inclusion criteria. We also conducted a risk of bias assessment for identified studies and found few examples of bias in the studies included in the evidence synthesis for scale-up (Figs 2 and 3). Our review provides a robust description of the scale-up terminology used in the studies that heretofore has not been reported. Finally, because there were no restrictions on article publication date, we captured a broad range of literature that describes the current state of scale-up terminologies within the implementation science field.

## Conclusion

Although this review failed to identify interventions that were brought to full scale for hypertension control in LMICs, it highlights the limited available data on intervention scalability for hypertension control in LMICs and demonstrates the need for scale-up metrics and processes for resource-constrained settings. The findings thus set the stage for a valid, reliable, and reproducible set of metrics for assessing scale-up potential of EBIs for HTN and other conditions.

## Supporting information

S1 File Appendix A: Search strategy. (DOCX)

S2 File PRISMA 2020 checklist. (DOCX)

## References

[1] Schutte A.E., et al., Hypertension in low-and middle-income countries. Circulation research, 2021. 128(7): p. 808–826. doi: 10.1161/CIRCRESAHA.120.318729 33793340 PMC8091106

[2] WHO., Raised blood pressure: Situations and trends. 2016.

[3] Mendis S., et al., Cardiovascular risk management and its impact on hypertension control in primary care in low-resource settings: a cluster-randomized trial. Bull World Health Organ, 2010. 88(6): p. 412–9. doi: 10.2471/BLT.08.062364 20539854 PMC2878142

[4] Ogedegbe G., et al., Task shifting interventions for cardiovascular risk reduction in low-income and middle-income countries: a systematic review of randomised controlled trials. BMJ Open, 2014. 4(10): p. e005983. doi: 10.1136/bmjopen-2014-005983 25324324 PMC4202019

[5] Mendis S., et al., Barriers to management of cardiovascular risk in a low-resource setting using hypertension as an entry point. J Hypertens, 2004. 22(1): p. 59–64. doi: 10.1097/00004872-200401000-00013 15106795

[6] Bosu W.K., Epidemic of hypertension in Ghana: a systematic review. BMC Public Health, 2010. 10: p. 418. doi: 10.1186/1471-2458-10-418 20626917 PMC2910685

[7] Gyamfi J., et al., Training nurses in task-shifting strategies for the management and control of hypertension in Ghana: a mixed-methods study. BMC Health Serv Res, 2017. 17(1): p. 104. doi: 10.1186/s12913-017-2026-5 28148255 PMC5288999

[8] Kayima J., et al., Hypertension awareness, treatment and control in Africa: a systematic review. BMC Cardiovasc Disord, 2013. 13: p. 54. doi: 10.1186/1471-2261-13-54 23915151 PMC3750220

[9] WHO/Expandnet, Nine-steps for Developing a scaling-up strategy. 2010.

[10] Glasgow R.E. and Emmons K.M., How can we increase translation of research into practice? Types of evidence needed. Annu Rev Public Health, 2007. 28: p. 413–33. doi: 10.1146/annurev.publhealth.28.021406.144145 17150029

[11] Milat A.J., et al., The concept of scalability: increasing the scale and potential adoption of health promotion interventions into policy and practice. Health Promot Int, 2013. 28(3): p. 285–98. doi: 10.1093/heapro/dar097 22241853

[12] Higgins, J., et al. Chapter 8: Assessing risk of bias in a randomized trial. In: Higgins JPT, Thomas J, Chandler J, Cumpston M, Li T, Page MJ, Welch VA (editors). Cochrane Handbook for Systematic Reviews of Interventions version 6.2 (updated February 2021). Cochrane, 2021. Available from: www.training.cochrane.org/handbook.

[13] Moher D., et al., Preferred reporting items for systematic reviews and meta-analyses: the PRISMA statement. J Clin Epidemiol, 2009. 62(10): p. 1006–12. doi: 10.1016/j.jclinepi.2009.06.005 19631508

[14] Group W.B., World development indicators 2014: World Bank Publications. 2014.

[15] WHO/ExpandNet, ExpandNet—Advancing the science and practice of scale up. 2010.

[16] Aira T., et al., Reducing risk behaviors linked to noncommunicable diseases in Mongolia: a randomized controlled trial. Am J Public Health, 2013. 103(9): p. 1666–74. doi: 10.2105/AJPH.2012.301175 23865647 PMC3780670

[17] Bekiroglu T., et al., Effect of Turkish classical music on blood pressure: a randomized controlled trial in hypertensive elderly patients. Complement Ther Med, 2013. 21(3): p. 147–54. doi: 10.1016/j.ctim.2013.03.005 23642944

[18] Chen Y., et al., A randomized controlled trial of the effects of brief mindfulness meditation on anxiety symptoms and systolic blood pressure in Chinese nursing students. Nurse Educ Today, 2013. 33(10): p. 1166–72. doi: 10.1016/j.nedt.2012.11.014 23260618

[19] Danaoglu Z., et al., Effect of statin therapy added to ACE-inhibitors on blood pressure control and endothelial functions in normolipidemic hypertensive patients. Anadolu Kardiyol Derg, 2003. 3(4): p. 331–7. 14675884

[20] Delavar F., Pashaeypoor S., and Negarandeh R., The effects of self-management education tailored to health literacy on medication adherence and blood pressure control among elderly people with primary hypertension: A randomized controlled trial. Patient Educ Couns, 2020. 103(2): p. 336–342. doi: 10.1016/j.pec.2019.08.028 31451361

[21] Forrester T., et al., A randomized trial on sodium reduction in two developing countries. J Hum Hypertens, 2005. 19(1): p. 55–60. doi: 10.1038/sj.jhh.1001782 15470483

[22] Gamage D.G., et al., Effectiveness of a scalable group-based education and monitoring program, delivered by health workers, to improve control of hypertension in rural India: A cluster randomised controlled trial. PLoS Med, 2020. 17(1): p. e1002997. doi: 10.1371/journal.pmed.1002997 31895945 PMC6939905

[23] Gong J., et al., Persistent effect at 30-month post intervention of a community-based randomized trial of KM2H(2) in reducing stroke and heart attack among senior hypertensive patients. Int J Behav Nutr Phys Act, 2018. 15(1): p. 1. doi: 10.1186/s12966-017-0635-3 29291739 PMC5749024

[24] Hacihasanoğlu R. and Gözüm S., The effect of patient education and home monitoring on medication compliance, hypertension management, healthy lifestyle behaviours and bmi in a primary health care setting. Journal of Clinical Nursing, 2011. 20(5/6): p. 692–705. 21320198 10.1111/j.1365-2702.2010.03534.x

[25] He J., et al., Effect of a Community Health Worker-Led Multicomponent Intervention on Blood Pressure Control in Low-Income Patients in Argentina: A Randomized Clinical Trial. JAMA, 2017. 318(11): p. 1016–1025. doi: 10.1001/jama.2017.11358 28975305 PMC5761321

[26] Huang B., et al., Effectiveness of self-management support in maintenance haemodialysis patients with hypertension: A pilot cluster randomized controlled trial. Nephrology (Carlton), 2018. 23(8): p. 755–763. doi: 10.1111/nep.13098 28666310

[27] Huang Y., et al., The effect of zolpidem on sleep quality, stress status, and nondipping hypertension. Sleep Med, 2012. 13(3): p. 263–8. doi: 10.1016/j.sleep.2011.07.016 22153779

[28] Jafar T.H., et al., A Community-Based Intervention for Managing Hypertension in Rural South Asia. N Engl J Med, 2020. 382(8): p. 717–726. doi: 10.1056/NEJMoa1911965 32074419

[29] Jafar T.H., et al., Community-based interventions to promote blood pressure control in a developing country: a cluster randomized trial. Ann Intern Med, 2009. 151(9): p. 593–601. doi: 10.7326/0003-4819-151-9-200911030-00004 19884620

[30] Jafar T.H., et al., Community based lifestyle intervention for blood pressure reduction in children and young adults in developing country: cluster randomised controlled trial. BMJ, 2010. 340: p. c2641. doi: 10.1136/bmj.c2641 20530082 PMC2881949

[31] Khan M.A., et al., Enhanced hypertension care through private clinics in Pakistan: a cluster randomised trial. BJGP open, 2019. 3(1): p. bjgpopen18X101617-bjgpopen18X101617. doi: 10.3399/bjgpopen18X101617 31049404 PMC6480862

[32] Kolcu M. and Ergun A., Effect of a nurse‐led hypertension management program on quality of life, medication adherence and hypertension management in older adults: A randomized controlled trial. Geriatr Gerontol Int, 2020. 20(12): p. 1182–1189. doi: 10.1111/ggi.14068 33079474

[33] Li H., et al., Effect of traditional and integrative regimens on quality of life and early renal impairment in elderly patients with isolated systolic hypertension. Chin J Integr Med, 2010. 16(3): p. 216–21. doi: 10.1007/s11655-010-0216-y 20694775

[34] Li N., et al., The effects of a reduced-sodium, high-potassium salt substitute on food taste and acceptability in rural northern China. Br J Nutr, 2009. 101(7): p. 1088–93. doi: 10.1017/S0007114508042360 18710605

[35] Lin P.H., et al., A text messaging-assisted randomized lifestyle weight loss clinical trial among overweight adults in Beijing. Obesity (Silver Spring), 2014. 22(5): p. E29–37. doi: 10.1002/oby.20686 24375969

[36] Naser A.M., et al., Consequences of access to water from managed aquifer recharge systems for blood pressure and proteinuria in south-west coastal Bangladesh: a stepped-wedge cluster-randomized trial. Int J Epidemiol, 2020. 10.1093/ije/dyaa098 PMC8271187 32653912

[37] Nguyen H.L., et al., Culturally adaptive storytelling intervention versus didactic intervention to improve hypertension control in Vietnam- 12 month follow up results: A cluster randomized controlled feasibility trial. PLoS One, 2018. 13(12): p. e0209912. doi: 10.1371/journal.pone.0209912 30596749 PMC6312314

[38] Ogedegbe G., et al., Health insurance coverage with or without a nurse-led task shifting strategy for hypertension control: A pragmatic cluster randomized trial in Ghana. PLoS Med, 2018. 15(5): p. e1002561. doi: 10.1371/journal.pmed.1002561 29715303 PMC5929500

[39] Pedrosa R.P., et al., Effects of OSA treatment on BP in patients with resistant hypertension: a randomized trial. Chest, 2013. 144(5): p. 1487–1494. doi: 10.1378/chest.13-0085 23598607

[40] Sarfo F.S., et al., Phone-based intervention for blood pressure control among Ghanaian stroke survivors: A pilot randomized controlled trial. Int J Stroke, 2019. 14(6): p. 630–638. doi: 10.1177/1747493018816423 30465630

[41] Tian M., et al., A Cluster-Randomized, Controlled Trial of a Simplified Multifaceted Management Program for Individuals at High Cardiovascular Risk (SimCard Trial) in Rural Tibet, China, and Haryana, India. Circulation, 2015. 132(9): p. 815–24. doi: 10.1161/CIRCULATIONAHA.115.015373 26187183 PMC4558306

[42] Varleta P., et al., Mobile phone text messaging improves antihypertensive drug adherence in the community. J Clin Hypertens (Greenwich), 2017. 19(12): p. 1276–1284. 28941056 10.1111/jch.13098 PMC8031315

[43] Yilmaz S., et al., Comparison of alprazolam versus captopril in high blood pressure: a randomized controlled trial. Blood Press, 2011. 20(4): p. 239–43. doi: 10.3109/08037051.2011.553934 21288144

[44] Li X., et al., A WeChat-Based Self-Management Intervention for Community Middle-Aged and Elderly Adults with Hypertension in Guangzhou, China: A Cluster-Randomized Controlled Trial. Int J Environ Res Public Health, 2019. 16(21). doi: 10.3390/ijerph16214058 31652688 PMC6862068

[45] Ma C., et al., Evaluation of the effect of motivational interviewing counselling on hypertension care. Patient Education and Counseling, 2014. 95(2): p. 231–237. doi: 10.1016/j.pec.2014.01.011 24530144

[46] Charif A.B.Z., H. T. V; LeBlanc A.;Langlois L.;Wolfenden L.;Yoong, S. L.;Williams C. M.;Lepine R.;Legare F., Effective strategies for scaling up evidence-based practices in primary care: a systematic review. Implement Sci, 2017. 12(1): p. 139. doi: 10.1186/s13012-017-0672-y 29166911 PMC5700621

[47] Baskerville N.B., Liddy C., and Hogg W., Systematic review and meta-analysis of practice facilitation within primary care settings. Ann Fam Med, 2012. 10(1): p. 63–74. doi: 10.1370/afm.1312 22230833 PMC3262473

[48] Mills K.T., et al., Comparative effectiveness of implementation strategies for blood pressure control in hypertensive patients: a systematic review and meta-analysis. Annals of internal medicine, 2018. 168(2): p. 110–120. doi: 10.7326/M17-1805 29277852 PMC5788021

[49] van de Glind I., et al., Making the connection-factors influencing implementation of evidence supported and non-evaluated lifestyle interventions in healthcare: a multiple case study. Health Educ Res, 2015. 30(4): p. 521–41. doi: 10.1093/her/cyv020 26025211

[50] Potter C. and Brough R., Systemic capacity building: a hierarchy of needs. Health Policy Plan, 2004. 19(5): p. 336–45. doi: 10.1093/heapol/czh038 15310668

[51] Bulthuis S.E., et al., Factors influencing the scale-up of public health interventions in low- and middle-income countries: a qualitative systematic literature review. Health Policy Plan, 2020. 35(2): p. 219–234. doi: 10.1093/heapol/czz140 31722382 PMC7050685

[52] Yamey G., What are the barriers to scaling up health interventions in low and middle income countries? A qualitative study of academic leaders in implementation science. Global Health, 2012. 8: p. 11. doi: 10.1186/1744-8603-8-11 22643120 PMC3514334

[53] Karp S.M., et al., Breastfeeding initiation in the context of a home intervention to promote better birth outcomes. Breastfeeding Medicine, 2013. 8(4): p. 381–387. doi: 10.1089/bfm.2012.0151 23484671 PMC3726024

