# Supplementary material for: Assessing descriptions of scalability for hypertension control interventions implemented in low-and middle-income countries: A systematic review
Source: PLoS One. 2022 Jul 28;17(7):e0272071. doi: 10.1371/journal.pone.0272071 (PMC9333290; doi:10.1371/journal.pone.0272071)
Supplement: S1 File — (DOCX) [file pone.0272071.s001.docx]

**Appendix A**

**Search Strategy for Systematic Review**

Below is the strategy used for the search of Ovid databases. The strategy was modified to suit the format for the other database search engines, i.e., Web of Science, Biosis Citation Index (BCI), CINAHL, EMBASE, Global Health, and PsycInfo. The search strategy below reflects modifications made to the strategy to address scale-up using title, abstract and subject heading field searching (see examples below the primary search strategy).

| (Elevated blood pressure OR hypertension OR hypertensions OR high blood pressure OR high blood pressures OR blood pressure determination OR blood pressure monitoring) AND (developing countries OR developing country OR LMIC OR low income countries OR low income country OR middle income countries OR middle income country OR third world country OR third world countries OR Africa OR Central Asia OR Western Asia OR Southeastern Asia OR Indian Ocean Islands OR Central America OR South America OR Eastern Europe OR Transcaucasia OR China OR Korea OR Mongolia OR Mexico OR Caribbean Region OR Pacific Islands OR Africa OR Central Asia OR Western Asia OR Southeastern Asia OR Indian Ocean Islands OR Central America OR South America OR Eastern Europe OR Transcaucasia OR Caribbean OR Pacific Islands OR Afghan OR Afghani OR Afghanistan OR Bangladesh OR Bangladeshi OR Benin OR Beninese OR Burkina Faso OR Burkinabe OR Burundi OR Burundian OR Cambodia OR Cambodian OR Central African Republic OR Central African OR Chad OR Chadian OR Comoros OR Comoran OR Congo OR Congolese OR Eritrea OR Eritrean OR Ethiopia OR Ethiopian OR Gambia OR Gambian OR Guinea OR Guinean OR Haiti OR Haitian OR Kenya OR Kenyan OR Korea OR Korean OR Kyrgyz OR Kyrgyzstan OR Liberia OR Liberian OR Madagascar OR Malagasy OR Malawi OR Malawian OR Mali OR Malian OR Mozambique OR Mozambican OR Myanmar OR Myanmarese OR Burmese OR Nepal OR Nepalese OR Niger OR Nigerian OR Rwanda OR Rwandan OR Sierra Leone OR Sierra Leonean OR Somalia OR Somalian OR Tajikistan OR Tajik OR Tadzhik OR Tanzania OR Tanzanian OR Togo OR Togolese OR Uganda OR Zimbabwe OR Zimbabwean OR Angola OR Angolan OR Armenia OR Armenian OR Belize OR Belizean OR Bhutan OR Bhutanese OR Bolivia OR Bolivian OR Cameroon OR Cameroonian OR Cape Verde OR Cape Verdian OR Cape Verdean OR Ivory Coast OR Djibouti OR Egypt OR Egyptian OR El Salvador OR Salvadoran OR Fiji OR Fijian OR Georgia OR Georgian OR Ghana OR Ghanaian OR Guatemala OR Guatemalan OR Guyana OR Guyanese OR Honduras OR Honduran OR Indonesia OR Indonesian OR India OR Indian OR Iraq OR Iraqi OR Kiribati OR Kosovo OR Kosovar OR Laos OR Lao OR Laotian OR Lesotho OR Marshall Islands OR Marshallese OR Mauritania OR Mauritanian OR Micronesia OR Micronesian OR Moldova OR Moldovan OR Mongolia OR Mongolian OR Morocco OR Moroccan OR Nicaragua OR Nicaraguan OR Nigeria OR Nigerian OR Pakistan OR Pakistani OR Papua New Guinea OR Papua New Guinean OR Paraguay OR Paraguayan OR Philippines OR Filipino OR Samoa OR Samoan OR Sao Tome OR Principe OR Santomean OR Senegal OR Senegalese OR Solomon Islands OR Solomon Islander OR Sri Lanka OR Sri Lankan OR Sudan OR Sudanese OR Swazi OR Swaziland OR Syria OR Syrian OR East Timor OR East Timorese OR Tonga OR Tongan OR Turkmenistan OR Turkmen OR Tuvalu OR Tuvaluan OR Ukraine OR Ukrainian OR Uzbekistan OR Uzbek OR Vanuatu OR Vietnam OR Vietnamese OR West Bank OR Gaza OR Yemen OR Yemeni OR Yemenite OR Zambia OR Zambian OR Albania OR Albanian OR Algeria OR Algerian OR Antigua and Barbuda OR Antiguan OR Barbudan OR Argentina OR Argentinian OR Azerbaijan OR Azerbaijani OR Belarus OR Belarusian OR Bosnia OR Bosnian OR Botswana OR Brazil OR Brazilian OR Bulgaria OR Bulgarian OR Barbados OR Bajan OR Barbadians OR Chile OR Chilean OR China OR Chinese OR Colombia OR Colombian OR Costa Rica OR Costa Rican OR Cuba OR Cuban OR Dominica OR Dominican OR Ecuador OR Ecuadorean OR Gabon OR Grenada OR Grenadian OR Iran OR Iranian OR Jamaica OR Jamaican OR Jordan OR Jordanian OR Kazakhstan OR Kazakhstani OR Latvia OR Latvian OR Lebanon OR Lebanese OR Libya OR Libyan OR Lithuania OR Lithuanian OR Macedonia OR Macedonian OR Malaysia OR Malaysian OR Maldives OR Maldivian OR Mauritius OR Mauritian OR Mexico OR Mexican OR Montenegro OR Montenegrin OR Namibia OR Namibian OR Palau OR Palauan OR Panama OR Panamanian OR Peru OR Peruvian OR Romania OR Romanian OR Russia OR Russian OR Serbia OR Serbian OR Seychelles OR Seychellois OR South Africa OR South African OR Saint Kitts OR Saint Lucia OR Saint Vincent OR Suriname OR Surinamer OR Thailand OR Thai OR Tunisia OR Tunisian OR Turkey OR Turkish OR Uruguay OR Uruguayan OR Venezuala OR Venezualan) AND (scalability.ti,ab. OR scale.ti,ab. OR scale-up.ti,ab. OR scalable.ti,ab. OR transferability.ti,ab. OR scale-out.ti,ab.) AND (Randomized Control Trials OR random OR randomization OR randomized OR randomized controlled trial OR randomized control) |
| --- |

Ovid databases strategy (Embase, Global Health and APA PsycInfo:

(scalability.ti,ab. OR scale.ti,ab. OR scale-up.ti,ab. OR scalable.ti,ab. OR transferability.ti,ab. OR scale-out.ti,ab.)

PubMed database:

(scalability [tw] OR scale [tw] OR scale-up [tw] OR scalable [tw] OR transferability [tw] OR scale-out [tw])

Field searching in the Title and Abstracts was conducted in the Web of Science and CINAHL databases.
